# Evaluation of Lactobacillus Coryniformis K8 Consumption by Health Care Workers Exposed to COVID-19 (LactoCor2 Project): Protocol for a Randomized Controlled Trial

**DOI:** 10.2196/37857

**Published:** 2023-06-28

**Authors:** Raquel Rodríguez-Blanque, Juan Carlos Sánchez-García, Angel Cobos Vargas, M Socorro Leyva Martínez, Silvia Martínez Diz, Jonathan Cortés-Martín, María Isabel Tovar-Gálvez

**Affiliations:** 1 Grupo de Investigación CTS-1068 Departamento de Enfermería, Facultad de Ciencias de la Salud, Universidad de Granada Granada Spain; 2 Hospital Universitario Clínico San Cecilio Granada Spain; 3 Grupo de Investigación CTS-1068 Hospital Universitario Clínico San Cecilio Granada Spain

**Keywords:** COVID-19, coronavirus, Lactobacillus, health care workers, Lactobacilo, trabajadores de la salud, SARS-CoV-2, probiótico

## Abstract

**Background:**

*Lactobacillus coryniformis* K8 CECT5711 has immune-modulating properties, enhances the immune response to viral antigens leading to the production of specific antibodies, and has anti-inflammatory activity, which may help to prevent uncontrolled inflammatory processes leading to respiratory and other organ failures.

**Objective:**

The purpose of this study is to evaluate the effect of the consumption of a probiotic strain on the incidence and severity of COVID-19 in health personnel who carry out their professional work among patients with infection or suspected infection by SARS-CoV-2.

**Methods:**

This is a double-blind randomized clinical trial in which the experimental group will receive a capsule of *L coryniformis* K8 per day (3×10^9^ colony former units/day), and the control group will receive a daily placebo capsule consisting of maltodextrin. A sample size of 314 volunteers was calculated. Volunteers must meet the following inclusion criteria: older than 20 years and active health personnel caring for patients with COVID-19, including all professionals such as medical doctors, nurses, and caretakers at the 2 referral hospitals that treat patients with COVID-19. The main outcome of the clinical trial will be the incidence of symptomatic infection by SARS-CoV-2 in personnel who care for patients with suspected or confirmed COVID-19.

**Results:**

The study had to be extended to the 2 referral hospitals that treat patients with COVID-19 in the province of Granada (Andalusia, Spain); Hospital San Cecilio and Hospital Virgen de las Nieves. A total of 255 individuals met the inclusion criteria and were randomly assigned to one of the 2 groups.

**Conclusions:**

The results of this randomized controlled trial will provide valuable information regarding the administration of *L coryniformis* K8 against COVID-19, including whether there are fewer infectious processes due to this virus or, in case of occurrence, whether the disease is milder in participants taking the probiotic strain.

**Trial Registration:**

ClinicalTrials.gov NCT04366180; http://www.clinicaltrials.gov/ct2/show/NCT04366180

**International Registered Report Identifier (IRRID):**

RR1-10.2196/37857

## Introduction

Humans have consumed lactic bacteria throughout history, but it was at the beginning of the 20th century when this consumption was first related to beneficial properties for health [1]. From that moment on, multiple studies both in vitro and in vivo have shown the beneficial effects that the consumption of this type of bacteria, called probiotics, has on health. Effects such as the improvement of intestinal function [2,3], prevention of intestinal infections [4], and immunomodulation have been widely demonstrated [5]. In addition to these beneficial effects, the consumption of probiotics has been related to a reduction in the incidence and severity of respiratory infections [4,6].

Oral administration of lactic acid bacteria has been associated with an increase in innate and acquired immune responses [7,8]. The consumption of certain strains has been shown to induce an increase in IgA related to the anti-infective properties of probiotic bacteria in diarrhea [9,10]. Innate immunity is increased through an increase in the proportion and activity of phagocytic cells of the immune system, such as monocytes and neutrophils. The function of natural killer cells is also enhanced by the consumption of probiotic bacteria [11]. Activation of the innate immune system is essential, as it produces cytokines that will lead to more specialized activation of the specific immune response. It has been suggested that it is through this mechanism that probiotic bacteria activate the immune response, thus being able to function as adjuvants in vaccination processes [12].

The European Food Safety Agency (EFSA) has published guidelines on how studies should be conducted to demonstrate the effect of a probiotic or natural ingredient on the immune system. Among its recommendations is the use of vaccination protocols that demonstrate how the administration of the probiotic translates into an improvement in the response to an antigen [13]. Recently, a study carried out at the Center Institute of Food Science and Technology and Nutrition of the Spanish National Research Council demonstrated that the administration of *Lactobacillus coryniformis* K8 CECT5711 increases the response of specific antibodies against hepatitis A in a vaccination protocol against this virus [14]. In addition, a certain effect on memory T-helper lymphocytes was observed, which increased in the probiotic group with respect to baseline values (mean 660, SD 223 cells/µL vs mean 551, SD 213 cells/µL). These results highlight the potential of this probiotic strain to improve the immune response against viral antigens.

To evaluate this activity that boosts the immune response, another study was carried out in an older population. This population is more susceptible to infections due to the characteristic immunosenescence of age. A total of 98 older people residing in nursing homes participated in this study. The volunteers received a daily capsule of *L coryniformis* K8 CECT5711 (3×10^9^ colony former units/day) for 2 weeks prior to being vaccinated against the flu or a placebo. The percentage of seroconversion was higher in the group receiving the probiotic than in the control group (93.1% vs 72.7%; *P*=.04). During the months of October to April, the volunteers were monitored, and the incidence of local symptoms associated with respiratory infections (sore throat, cough, and nasal congestion) was 48% lower in the group that received the probiotic strain than in the control group (*P*=.007). Furthermore, the consumption of analgesics was significantly reduced in this group by 86% (*P*=.008) [15]. This latest study corroborates the strain’s ability to improve the immune response against viral antigens even in the older population.

We are currently experiencing a pandemic due to COVID-19 (the English acronym for COVID-19), which started in the Chinese city of Wuhan in December 2019. Having reached more than 100 territories, the disease was declared a pandemic by the World Health Organization on March 11, 2020 [16]. The number of confirmed cases continued to grow until reaching 5,371,660 cases worldwide on May 22, 2020. In Spain, at the end of May 2020, despite the containment measures initiated in early March, more than 235,772 cases were registered, and of these patients, more than 27,940 died [17].

The COVID-19 infection produces flu-like symptoms, including fever, dry cough, dyspnea, myalgia, and fatigue. In severe cases, it is characterized by pneumonia, acute respiratory distress syndrome, and septic shock, which lead to death in approximately 3% of those infected. Transmission occurs through small droplets that are emitted when speaking, sneezing, or coughing by a carrier and pass directly to another person through inhalation or by contact with contaminated objects in the recipient’s nasal and ocular mucous membranes. Symptoms start after approximately 7-20 days of exposure, and prevention at the population level consists of frequent hand washing, disinfecting surfaces with alcohol, and avoiding close contact with other people [15].

Some drugs have been proposed as antiviral treatments, such as hydroxychloroquine, lopinavir, ritonavir, and others; severe pneumonia is treated with broad-spectrum antibiotics. There is no up-to-date, effective treatment for the COVID-19 infection.

An article published in March 2020 shows how the immune response to the COVID-19 virus is similar to the response generated by the influenza virus [18]. Some authors propose the use of probiotics as a strategy to mitigate the effects of viruses [19].

Health care workers (HCWs) are at increased risk of COVID-19 infection due to close contact with highly infectious patients as well as exposure to undiagnosed or subclinical infected cases. The stress and physical exhaustion associated with the pandemic may also impact the immune response of HCWs [20]. A recent study examined 97 studies and found that approximately 11% of patients with COVID-19 were people with underlying disease [21]. This represented a considerable percentage of all COVID-19 cases [22]. Although the number of severe cases and mortality among HCWs was lower than in other populations [21], the number of COVID-19 cases has led to a shortage of HCWs, thus adding an extra burden to the fight against this pandemic.

It has been suggested by several authors that probiotics could play a role in preventing and alleviating the severity of COVID-19 due to their immune-modulating, anti-inflammatory, antioxidant, and antiviral properties [23-25]. The *Loigolactobacillus coryniformis* K8 CECT 5711 strain has been shown to have the immune-modulating capacity in adults [26] and children [27,28]. Research has shown that this strain causes an increase in hepatitis A virus antibody levels after oral administration in healthy adults as part of hepatitis A vaccination [14]. A study in older people found that the administration of *L coryniformis* K8 strengthened the immune response to influenza vaccination and reduced symptoms associated with respiratory infections [15]. A recent randomized controlled trial conducted with nursing home residents showed the benefits of *L coryniformis* K8 in the COVID-19 pandemic. This is because it may enhance the specific immune response following COVID-19 infection and also aid vaccine-specific responses among the older population [29].

In this context, it is proposed that the immunomodulatory properties of *L coryniformis* K8 CECT5711 might be useful for COVID-19 management.

## Methods

### Aim of the Study

The objective of this study is to evaluate the effects of the consumption of *L coryniformis* K8 on the incidence and severity of symptomatic infection by SARS-CoV-2 and the severity of COVID-19 in HCWs who carry out their professional work among patients with infection or suspected infection by SARS-CoV-2 at 2 referral hospitals that treat individuals with COVID-19 in the province of Granada (Andalusia, Spain).

### Description of Treatments

The administration of the experimental product and the placebo will be carried out in the form of a gelatin capsule that will have the same weight, consistency, and color.

The Experimental Group will receive hard gelatin capsules that will contain 3×10^9^ colony forming units of *L* *coryniformis* K8 (120 mg), 10 mg magnesium stearate, and 120 mg maltodextrin.

The control group will receive hard gelatin capsules that will contain magnesium stearate (10 mg) and maltodextrin (220 mg). Individuals from both groups will take 1 capsule a day (preferably with dinner).

Capsules for the probiotic and placebo will be provided by Biosearch Life (Granada, Spain).

### Study Design

**Figure 1:**
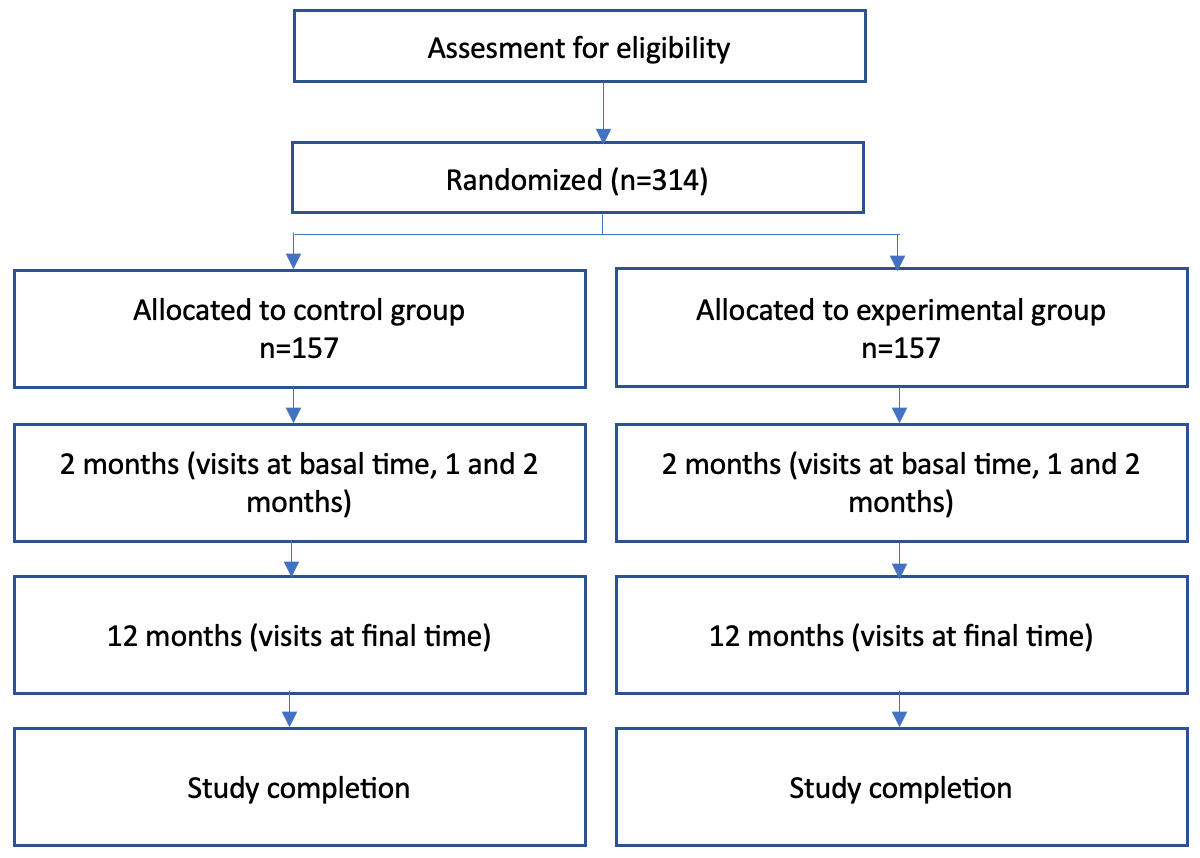
Study flowchart.

The study is a nutritional, randomized, double-blind, controlled, and parallel-group observational prospective cohort study. Volunteers will be distributed into 2 groups: a control group and an experimental group. The study will be performed as shown in Figure 1.

The trial has been registered on ClinicalTrials.gov (NCT04366180).

### Ethics Approval

This clinical research protocol has been prepared with full understanding and in accordance with the standards of Good Clinical Practice in accordance with CPMP/ICH/135/95, the Declaration of Helsinki (64th General Assembly, Fortaleza, Brazil, October 2013), ISO 14155, and all relevant national guidelines. All investigators participating in the study are appropriately qualified. The protocol was approved by the Research Ethics Committee for the province of Granada (3/2020_P_054; CEIM/CEI Province of Granada). Volunteers will be included in the study after informed written consent is obtained.

### Participants, Intervention, and Outcomes

#### Inclusion Criteria

The inclusion criteria included individuals older than 18 years and active HCWs, including all professional categories such as medicine, nursing, and caretaking, who attend cases classified as positive or suspicious for COVID-19 in the emergency department, intensive care unit (ICU), or in the wards designated to care for such patients; and the ability to complete the surveys and having signed the informed consent.

#### Exclusion Criteria

The exclusion criteria excludes individuals with a positive test for COVID-19 confirmed by polymerase chain reaction or serology, individuals with concomitant pathology such as HIV, transplantation, active cancer, or other active immunosuppression, and women who are pregnant or intend to become pregnant in the subsequent months.

#### Recruitment

The recruitment of participants will take place at the San Cecilio University Clinical Hospital and at the Virgen de las Nieves University Hospital in Granada among HCWs with high exposure to patients with COVID-19.

#### Randomization, Allocation, and Blinding

Patients will be randomly allocated to a control group or experimental group in a 1:1 ratio by the block randomization method with a block size of 4. Participants and investigators will be blinded to the treatment allocation during the course of the intervention. The blind will be broken after statistical analysis is completed.

Capsules for the control and experimental groups will be provided in white containers labeled with a random number.

#### Intervention

Participants meeting the inclusion criteria will be randomly assigned to the control or experimental group. During the intervention, participants will be asked to ingest a capsule per day with some food (preferably with dinner). Follow-up visits will be scheduled at the beginning, midpoint (1 month), and end of the intervention period (2 months). At the first visit, baseline data (age, sex, lifestyle, medication, concomitant diseases, and current symptoms) will be collected. At follow-up visits, data about symptoms, medication, and adverse effects will be recorded (Table 1).

**Table 1:** Detailed study procedures.

Visits (V) per week (W)	V1 (W0)	V2 (W4)	V3 (W8)	V4 (12 months)
Informed consent form	✓			
Inclusion or exclusion criteria	✓			
Randomization or allocation	✓			
Treatment distribution	✓			
Basal data	✓			
COVID-19 test	✓	✓^a^	✓	
Symptoms questionnaire		✓	✓	
Medication questionnaire	✓	✓	✓	
Adverse effects questionnaire		✓	✓	
Return of unconsumed capsules			✓	
Evaluation of the immune response to the vaccine				✓

^a^COVID-19 test data would be collected only if performed.

### Outcomes Measures

#### Primary Outcomes

The main outcome will be the incidence of symptomatic SARS-CoV-2 infection confirmed by polymerase chain reaction or serology in HCW caring for patients with suspected or confirmed COVID-19 at the San Cecilio Clinical University Hospital and at the Virgen de las Nieves University Hospital.

#### Secondary Outcomes

Incidence of hospital admissions caused by SARS-CoV-2 infection, the incidence of ICU admissions caused by SARS-CoV-2 infection, the incidence of pneumonia caused by SARS-CoV-2 infection, the incidence of the need for oxygen support, the incidence of gastrointestinal symptoms, days with a body temperature of >37.5 °C, days of persistent cough, days of persistent feeling of fatigue, and use of pharmacological treatments. Data for mild symptoms will be recorded daily in the notebook of the volunteer, and, in the case of hospitalization, data will be collected by investigators from medical histories.

In addition, the immune response and side effects of the COVID-19 vaccine in these HCWs will be assessed. Data will be collected on the vaccines received by the HCWs, including the name and type of vaccine, doses received, date of vaccination, and immune response of the HCWs.

### Withdrawal, Dropout, Discontinuation, and Compliance

Withdrawal will be allowed at any time during the trial. Investigators may recommend discontinuing the trial if, in their opinion, staying in the study would be detrimental to the participant’s well-being. This decision must be based on the appearance of an adverse effect that will be duly included in the case report form.

Volunteers will return the unconsumed capsules. Compliance ≤80% of the total will be considered a violation of the protocol and will be considered dropouts.

### Adverse Effects

*L coryniformis* species are included in the Qualified Presumption of Safety list published by the EFSA, which guarantees safety for consumption [30]. On each visit, it will be monitored for adverse effects, which will be recorded in the patient’s medical history and evaluated by the principal investigator. In the event of serious adverse events considered related to the experimental product, the researcher will inform the health authorities and the Clinical Research Ethics Committee involved in the clinical trial.

### Sample Size

Given that the current rates of infection among health professionals worldwide are very different, the sample size has been based on the comparison of 2 independent proportions for the use of the chi-square test. For an α of 5% and a power of 80%, taking a percentage of affected individuals in the control group of 15% and 10% as the minimum meaningful difference to be detected, a sample of 157 per branch would be necessary, taking into account a loss of 10% of participants.

### Statistical Analysis

Due to the urgency of the circumstances of the pandemic, the statistical methods have not been fully planned. A statistical analysis plan will be prepared during the execution of the study and before the closure of the database. The statistical methods to be used, the strategy to be followed in the case of missing values, and the tables and figures to include in the statistical report will be described in detail.

The sample size has been calculated assuming 10% dropouts. It is not planned to replace withdrawal participants. Intention-to-treat analysis will be carried out, taking into account all the data of the recruited volunteers, even if they have not completed the intervention.

In general, the data will be presented as absolute frequencies and percentages in the case of qualitative variables. For the quantitative variables, central tendency statistics, such as the mean and median, and dispersion statistics, such as the SD, IQR, minimum and maximum values, and to indicate the shape of the distribution, shape measures such as coefficients of skewness and kurtosis, will be used. In the case of ordinal variables, depending on the number of categories, one form or another of description will be used. In all cases, a column that includes the number of volunteers with available data must be added to the data presentation table. The analyses will be divided by randomization group.

To analyze the comparisons between groups, parametric tests (Student *t* test or ANOVA) and nonparametric tests (Mann-Whitney *U* or Kruskal-Wallis) will be used for continuous variables according to the characteristics of the study variables (assumption normality), while for categorical variables, chi-square tests or Fisher exact tests will be used.

In the case of between-visit comparisons of continuous variables, parametric tests (Student *t* test for paired data or repeated measures ANOVA) and nonparametric tests (Wilcoxon or Friedman) will be used, as appropriate, according to the characteristics of the study variables (assumption of normality) and the number of visits to be compared, while the categorical variables will be compared using the McNemar test. The effect size will be estimated using CIs.

The significance level of the statistical tests used will be .05. All calculations will be performed with the SPSS statistical software package (version 26; IBM Corp).

For the analysis of adverse events, a descriptive analysis (frequencies and percentages) will be carried out throughout the study, presenting a list of the adverse events grouped according to severity. These events will be listed by volunteer and randomization groups.

Adverse events and concomitant medication will be coded using a standardization system. In the case of adverse events, the most current version of the MedDRA system will be used, and for concomitant medication, the ATC code will be used.

### Data Management

To further ensure the accuracy and reliability of the collected data, a thorough process of data cleansing will be implemented before computerization at the coordinating center in Granada, Spain. Any errors or inconsistencies identified during this process will be rectified or clarified with the relevant source documents or database managers. The source documents and databases will also be subjected to regular data cleansing procedures to maintain their integrity throughout the minimum 5-year retention period after the study’s conclusion. Additionally, any personal information will be carefully screened and removed as part of the data cleansing process to guarantee anonymity and confidentiality.

### Dissemination

Communications and scientific reports that emerge from this study will be carried out under the responsibility of the principal investigator in agreement with the associated investigators. Publication rules will follow international recommendations. The findings will also be shared with national health authorities.

Authorship will follow the guidelines established by the International Committee of Medical Journal Editors [31], which require substantive contributions to the design, conduct, interpretation, and reporting of a trial.

## Results

The study had to be extended to the 2 referral hospitals that treat COVID-19 cases in the province of Granada (Andalusia, Spain), Hospital San Cecilio and Hospital Virgen de las Nieves because the projected recruitment was not sufficient for the objective of the study and it was necessary to expand the sample obtained in order to reach as close as possible to the calculated sample. Finally, 255 volunteers were recruited and randomly distributed into 2 groups: the control group (n=128) and the probiotic group (n=127). In order to measure additional secondary outcomes, the project was kept open by collecting data related to vaccines and immune responses. The study started in July 2020, with the first part of the study ending in September 2020. In order to measure additional secondary outcomes, the project was kept open by collecting data related to vaccines and immune responses until September 2021. We will start analyzing responses in July 2023, with publication of results expected in the second half of 2023.

## Discussion

### Principal Findings

This study is conducted to evaluate the efficacy of the effects of *L coryniformis* K8 consumption on the incidence and severity of COVID-19 in HCWs working with SARS-CoV-2–infected or suspected patients in referral hospitals for patients with COVID-19 in the province of Granada. This randomized controlled trial aims to study whether the consumption of *L coryniformis* K8 will reduce the incidence of SARS-CoV-2 infection in the staff caring for patients with suspected or confirmed COVID-19 disease at the San Cecilio University Hospital and Virgen de las Nieves University Hospital. For this purpose, the incidence of hospital admissions caused by SARS-CoV-2 infection, both in the general hospitalization area and in the ICU area, will be studied in HCWs caring for SARS-CoV-2–infected patients.

It is important to determine the prevalence of pneumonia caused by SARS-CoV-2 infection and the need for oxygen supply to these patients. The presence of fever will be studied, and the number of days will be quantified, as well as the persistence of cough and feeling of fatigue. On the other hand, it will also be assessed whether these workers have needed pharmacological treatment.

This intervention aims to shed light on the use of probiotics to enhance the immune effect against SARS-CoV-2. In addition, this intervention study aims to contribute to the understanding of the effect of taking probiotics to improve the immune response to vaccines against SARS-CoV-2. An article published in March 2020 shows how the immune response to the COVID-19 virus is similar to the response generated by the influenza virus [18]. When the immune system works properly, the first barrier to SARS-CoV-2 is the innate response. This innate response, characterized by interferon and cytokines, which are small proteins responsible for symptoms such as fever, headaches, and muscle pain, has 2 functions: to slow down the replication and spread of the virus until the specific immune response (which, when it is a virus that has not been previously contacted, takes approximately 2 to 3 weeks) is activated. The specific immune response is what will stop and ultimately eliminate the infection. This specific response also controls the innate response in order to prevent excessive activity from damaging the tissues. When this response works in a correct and coordinated manner, the infection is overcome, showing symptoms more or less similar to those of the flu. However, in an older population experiencing dysfunction of the age-related immune response, called immunosenescence, or in a population in which the response appears to be poorly coordinated, the innate response occurs later or is ineffective, giving the virus time to replicate and spread and delaying the activation of the specific response. Before the massive invasion of tissues, the innate response exaggeratedly damages tissues, causing respiratory failure and failure in other organs, such as the kidney. Given this situation, some authors speak of 3 disease states that require different approaches: an asymptomatic state, a second state with nonsevere symptoms, and a third state with severe respiratory symptoms and a high viral load. During the first 2 states, a specific immune response is required to eliminate the virus and prevent the disease from progressing to state 3. In this phase, strategies to enhance the immune response are important. However, in state 3, when the immune system has failed and the virus has spread, the damage to the tissues causes a massive inflammatory response; the strategy should be aimed at controlling that inflammation [27]. Several clinical trials have evidenced the positive effects of certain probiotic strains on the prevention of respiratory tract infections. In fact, some authors propose the use of probiotics as a strategy to mitigate the effects of SARS-CoV-2 [19]. Mechanisms that would be involved in a positive effect of probiotic treatments on respiratory viruses include enhancement of the intestinal epithelial barrier, competition with pathogens for nutrients and adhesion to the intestinal epithelium, production of antimicrobial substances, and modulation of the host immune system [19]. *L coryniformis* K8 CECT5711 has immunomodulatory properties that could be useful for COVID-19 management. On the one hand, we have previously discussed its ability to improve the immune response against viral antigens, leading to the production of specific antibodies [14,15]. On the other hand, the strain has been shown in animal experiments to have anti-inflammatory capacity in models of inflammation [3,27]. This anti-inflammatory activity would also be of interest in the case of COVID-19, as it could help prevent uncontrolled inflammatory processes from leading to respiratory and other organ failure.

The working hypothesis is that the administration of *L coryniformis* K8 will stimulate the immune response of people exposed to COVID-19, acting as an adjuvant that contributes to developing an effective response against the virus, and therefore fewer infectious cases are presented due to this virus or, in the event of an occurrence, the disease is milder among the participants taking the probiotic strain.

The high degree of exposure among HCWs puts them at high risk of contracting the virus. If we add to this situation the fact that the stress and physical overexertion caused by the pandemic can affect the immune response, the risk increases even more. The study has been designed to demonstrate if the consumption of these probiotic bacteria might contribute to reducing the level of incidence, or at least the severity of infection, in this population.

### Conclusions

Further knowledge in this area could lead to an improvement in the immune response of HCWs to noninfection with SARS-CoV-2 or to a reduction of symptoms in cases of infection. This would ensure that HCW workforces are not impaired in their ability to cope with SARS-CoV-2 and that those infected with SARS-CoV-2 are able to return to work more quickly than those who do not take probiotic supplements.

## Abbreviations

EFSA: European Food Safety Agency
HCW: health care worker
ICU: intensive care unit

## Data Availability

The data sets generated during this study are available from the corresponding author upon reasonable request.
